# Efficacy and Safety of Surgical Management for Cerebral Arteriovenous Malformations: A Study Involving 100 Cases

**DOI:** 10.7759/cureus.99887

**Published:** 2025-12-22

**Authors:** Karim N Zamora-Amezcua, Roberto Jesús Medellín Sánchez

**Affiliations:** 1 Neurological Surgery, Puerta de Hierro Andares Medical Center, Zapopan, MEX; 2 Vascular Neurosurgery, High Specialty Medical Unit No. 25, Mexican Social Security Institute (IMSS), Monterrey, MEX

**Keywords:** arteriovenous malformations, microsurgery, neurosurgical procedures, spetzler-martin, treatment outcome

## Abstract

Introduction: Cerebral arteriovenous malformations (AVMs) are congenital vascular anomalies that represent a significant surgical challenge due to their risk of bleeding and anatomical complexity. The Spetzler-Martin (SM) classification is a fundamental tool for predicting surgical risk and guiding treatment.

Objective: This study aimed to assess the clinical and functional outcome of microsurgical treatment in patients with AVMs according to their SM grading classification.

Methods: The present study employed a prospective, multicenter observational cohort of 100 consecutive patients with surgical resection of AVMs during January 2008 and May 2020 in four Mexico-based public and private hospitals. The AVMs were grouped based on the SM scale (grade I-V), and the data were taken at baseline, discharge, and six months after regarding the clinical, anatomical, and functional measures. The Glasgow Coma Scale (GCS) and the modified Rankin Scale (mRS) were taken as functional outcomes. The analysis of the data was based on descriptive statistics and nonparametric tests, specifically the Kruskal-Wallis test, with a significance level of 0.05.

Results: A total of 62 patients (62%) had grade I and III AVMs. Functional recovery was complete in 30 patients (30%) with grade I AVMs and favorable in 22 patients (22%) with grade II AVMs. Greater variability in outcomes was observed in grade III AVMs. Grades IV and V presented greater morbidity and less functional recovery. Inferential analysis showed significant differences between the groups on the Rankin scale (p < 0.001), but not on the GCS (p = 0.178). During the follow-up of five years, no recurrences, rebleeding events, or mortality related to malformations arteriovenous or with the surgical procedure, demonstrating the effectiveness and safety of the long-term microsurgical treatment.

Conclusions: The SM classification is useful for predicting postoperative functional outcomes. Surgical treatment is more effective and safer for low-grade AVMs and should be performed in centers with neurovascular expertise.

## Introduction

Cerebral arteriovenous malformations (AVMs) are congenital vascular anomalies characterized by a direct connection between arteries and veins without capillary interposition [1,2]. AVMs are a significant cause of bleeding in the brain, the population, and young adults, although AVMs are uncommon [3,4].

The abnormality of each AVM is the result of complications of varying patterns of blood flow, blood vessels, and overall shape of the brain region in which they are located [5,6]. AVM treatment ranges from doing nothing (observation) to radiosurgery, endovascular embolization, and microsurgery [7].

The varying degrees of Spetzler-Martin (SM) grading of the AVM, based on brain region, size, eloquence of the surrounding patient anatomy, and venous drainage, are used and have been shown to predict and still guide AVM treatment in the microsurgery era, especially SM II and III [8,9]. However, its application is not without controversy, especially in intermediate grades (II and III), where decisions must be individualized.

Despite advances in surgical techniques and intraoperative imaging, microsurgical resection remains the gold standard for accessible, low- to medium-grade AVMs, with cure rates exceeding 90% in specialized centers [10,11]. However, the risks of neurological deficit and postoperative complications persist, particularly in grade IV lesions or in deep locations [12]. This study attempts to deal with the lack of long-term data in Latin America regarding the microsurgical treatment of AVMs of all SM grades. Hence, this study aims to add region-informed data to evaluate the clinic and functional outcomes of AVMs of SM grades microsurgically treated in this region.

## Materials and methods

Study settings

The present study addresses the limited long-term data available in Latin-American populations undergoing microsurgical treatment of AVMs across all SM grades. Therefore, the objective of this study is to evaluate the clinical and functional outcomes of 100 microsurgically treated AVMs according to the SM classification, providing region-specific evidence for surgical decision-making.

A prospective, longitudinal, observational clinical study with a descriptive and inferential statistical approach was conducted in four institutions: two public hospitals: the High Specialty Medical Unit No. 25 of the Mexican Social Security Institute (IMSS) in Monterrey, Nuevo León, and the Specialty Hospital of the National Medical Center of the West of the IMSS in Guadalajara, Jalisco, as well as two private hospitals: Puerto de Hierro Andares Medical Center and Terranova Hospital, both located in Guadalajara, Jalisco. The study period ran from May 2008 to May 2020.

Study population and sampling strategy

Patients with a diagnosis of cerebral AVM, confirmed by cerebral angiography, who underwent surgical treatment between January 2008 and May 2020, were included. The authors of this study performed the surgical interventions: RJMS in Monterrey between 2008 and 2010, and KNZA, who served as first assistant in surgeries performed in Monterrey (2008-2010) and as primary surgeon in procedures performed in Guadalajara between 2010 and 2018.

Inclusion and exclusion criteria

Patients with cerebral AVMs of any sex and age who underwent surgery, had an angiographic diagnosis, and had a minimum of six months of clinical follow-up were included. Patients with an incomplete diagnosis, those who had undergone only endovascular treatment or radiosurgery, and those without a record of postoperative functional assessment were excluded.

Sample size calculation

To compute the minimal sample size required for compilation, we used outcome data from the available AVM surgical literature. Previously published randomized prospective studies have claimed a 6% incidence of permanently disabling neurological deficit after microsurgical resection of cerebral AVM, as described in the study by Hartman et al. [13]. With a 5% desired precision and 95% confidence level, the required minimal number of participants to ensure the desired precision was determined using the formula:

$N = \frac{Z^2 \, p(1 - p)}{d^2}$.

The calculated sample size was 87. To increase statistical reliability and therefore allow comparisons of meaningful subgroups by SM grade, we enrolled a large number of patients (n = 100) to attain the required analytic power in a number (n = 100) much higher than the minimum calculated.

Study instrument

Data were collected on age, sex, personal medical history (high blood pressure, smoking, alcoholism, and epilepsy), clinical presentation (headache, hemorrhagic stroke, seizures, and neurological impairment), anatomical location of the AVM, and laterality (right or left hemisphere).

AVMs were categorized according to the SM grading system, which assigns grades I to V based on lesion size, location in eloquent brain regions, and venous drainage pattern [8], and the results were evaluated using two functional scales: the Glasgow Coma Scale (GCS) and the modified Rankin Scale (mRS), at three times: preoperatively, upon hospital discharge, and at six months of follow-up.

Study process

All patients were treated with microsurgical resection, with individualized approaches based on the anatomical location and complexity of the lesion. The procedures were performed by the same neurosurgical team specializing in cerebrovascular surgery.

Statistical analysis

Statistical analysis was performed using descriptive and inferential statistics. Absolute and relative frequencies were used for categorical variables, and measures of central tendency and dispersion (mean, standard deviation) were used for numerical variables. Nonparametric tests (Kruskal-Wallis test) were used to compare groups according to the SM degree. A p value of <0.05 was considered statistically significant. Data processing was carried out using IBM Statistical Package for the Social Sciences Statistics version 26 software (IBM Corp., Armonk, NY).

Ethical clearance

The protocol for this study was approved by the Local Research Ethics Committee of the IMSS, in compliance with the regulations of the General Health Law on Health Research in Mexico. Furthermore, the study was conducted according to the ethical principles contained in the 1975 Declaration of Helsinki and its subsequent amendments.

The data were collected from institutional medical records, ensuring confidentiality, anonymity, and the protection of patients' personal information at all times. Data management was carried out in strict compliance with current ethical and regulatory provisions, without posing any direct risk to participants.

The protocol was registered with the Research Ethics Committee with folio number IMSS-R-2010-2501-XX. The present manuscript adheres to the STrengthening the Reporting of OBservational studies in Epidemiology guidelines for observational studies.

## Results

One hundred patients diagnosed with cerebral AVM who underwent surgical treatment between January 2008 and December 2020 were included. The sex distribution was equal, with 50 men (50%) and 50 women (50%). The mean age was 28.55 ± 13.4 years, with a range of 3-55 years (Table 1).

**Table 1:** General characteristics of patients with AVM included in the study SD: standard deviation; AVM: arteriovenous malformation

Variable	Worth
Number of patients	100
Sex	
Male	50 (50%)
Female	50 (50%)
Age, mean (±SD)	28.55 ± 13.4 years
Age range	3-55 years old

Among the personal histories recorded, 21 patients (21%) had epilepsy or seizures, 20 patients (20%) reported tobacco use, six patients (6%) had hypertension, and four patients (4%) had alcoholism. The personal medical histories of the cohort are summarized in Table 2.

**Table 2:** Personal history of patients with AVM AVM: arteriovenous malformation

Background	Frequency (%)
Epilepsy/seizures	21 (21%)
Smoking	20 (20%)
Hypertension	6 (6%)
Alcoholism	4 (4%)

In terms of clinical presentation, headache was present in 94 patients (94%), hemorrhagic stroke in 66 patients (66%), and seizures in 61 patients (61%). Furthermore, 47% of patients presented some degree of acute neurological deterioration at the time of admission. Clinical presentations at admission are detailed in Table 3.

**Table 3:** Clinical presentations of patients with AVM AVM: arteriovenous malformation

Clinical presentation	Number of patients (n)	Percentage (%)
Holocranial (diffuse) headache	94	94%
Hemorrhagic stroke	66	66%
Seizures	61	61%
Neurological deterioration on admission	47	47%

From an anatomical location perspective, cerebral AVMs occurred in 67 patients (67%) in the right hemisphere and 33 patients (33%) in the left hemisphere. Regarding cortical and subcortical topography, parietal lobe: 34 patients (34%), frontal: 23 (23%), cerebellum: 12 (12%), occipital: 11 (11%), temporal: 8 (8%). Additionally, AVMs were identified in intraventricular (four cases), thalamic (four cases), and insular (two cases) locations. The complete distribution is shown in Table 4.

**Table 4:** Anatomical distribution of AVMs according to hemisphere and topographic location AVM: arteriovenous malformation

Anatomical location	Right hemisphere, n (%)	Left hemisphere, n (%)	Total, n (%)
Frontal lobe	10 (10%)	6 (6%)	16 (16%)
Parietal lobe	26 (26%)	9 (9%)	35 (35%)
Occipital lobe	13 (13%)	4 (4%)	17 (17%)
Temporal lobe	11 (11%)	4 (4%)	15 (15%)
Cerebellum	8 (8%)	5 (5%)	13 (13%)
Intraventricular	4 (4%)	0 (0%)	4 (4%)
Thalamic	4 (4%)	0 (0%)	4 (4%)
Insular	1 (1%)	1 (1%)	2 (2%)
Total	67 (67%)	33 (33%)	100 (100%)

Based on the SM classification, AVMs were distributed as follows: 30 patients (30%) were grade I, 22 (22%) were grade II, 32 (32%) were grade III, 14 (14%) were grade IV, and two patients (2%) were grade V. This distribution included all grades surgically treated during the study period. The highest proportions were observed in grades I and III, which together accounted for 62% of the total cases analyzed (Table 5).

**Table 5:** Distribution of patients according to Spetzler-Martin grade

Spetzler-Martin degree	Number of patients (n)	Percentage (%)
Grade I	30	30.0%
Grade II	22	22.0%
Grade III	32	32.0%
Grade IV	14	14.0%
Grade V	2	2.0%
Total	100	100.0%

Functional assessment according to the SM grade

Grade I

Thirty patients with grade I AVM were included. At preoperative evaluation using the GCS, all patients (100%) had a score of 15, corresponding to level I. At hospital discharge, 28 patients maintained this score, while two dropped to level II and four to level III. However, at the six-month evaluation, all patients had recovered to the full score (level I), with no evidence of neurological deficit, as shown in Table 6.

**Table 6:** Functional evaluation of patients with cerebral AVM according to Spetzler-Martin grade (n = 100) GCS: Glasgow Coma Scale; mRS: Modified Rankin Scale; AVM: arteriovenous malformation

Grade	Assessment	Level	Preoperative, n (%)	Discharge, n (%)	6 month, n (%)
I (n = 30)	GCS	I	30 (100%)	28 (93.3%)	30 (100%)
		II	0 (0%)	2 (6.7%)	0 (0%)
		III	0 (0%)	0 (0%)	0 (0%)
	mRS	0	30 (100%)	29 (96.7%)	30 (100%)
		1	0 (0%)	1 (3.3%)	0 (0%)
		2	0 (0%)	0 (0%)	0 (0%)
II (n = 22)	GCS	I	17 (77.3%)	16 (72.7%)	22 (100%)
		II	5 (22.7%)	2 (9.1%)	0 (0%)
		III	0 (0%)	4 (18.2%)	0 (0%)
	mRS	0	3 (13.6%)	0 (0%)	16 (72.7%)
		1	12 (54.5%)	15 (68.2%)	5 (22.7%)
		2	6 (27.3%)	2 (9.1%)	1 (4.5%)
		3	1 (4.5%)	4 (18.2%)	0 (0%)
		4	0 (0%)	1 (4.5%)	0 (0%)
III (n = 32)	GCS	I	24 (75.0%)	18 (56.3%)	30 (93.8%)
		II	5 (15.6%)	8 (25.0%)	0 (0%)
		III	2 (6.3%)	5 (15.6%)	1 (3.1%)
		IV-V	1 (3.1%)	1 (3.1%)	1 (3.1%)
	mRS	0	1 (3.1%)	0 (0%)	21 (65.6%)
		1	23 (71.9%)	18 (56.3%)	8 (25.0%)
		2	2 (6.3%)	4 (12.5%)	1 (3.1%)
		3-4	6 (18.7%)	7 (21.9%)	2 (6.3%)
IV (n = 14)	GCS	I	6 (42.9%)	3 (21.4%)	9 (64.3%)
		II	5 (35.7%)	5 (35.7%)	2 (14.3%)
		III-V	3 (21.4%)	6 (42.9%)	3 (21.4%)
	mRS	0-1	6 (42.9%)	4 (28.6%)	9 (64.3%)
		2-3	6 (42.9%)	6 (42.9%)	4 (28.6%)
		4	2 (14.3%)	2 (14.3%)	1 (7.1%)
V (n = 2)	GCS	III	2 (100%)	2 (100%)	2 (100%)
	mRS	4	2 (100%)	2 (100%)	2 (100%)

Regarding the mRS, all patients were at level 0 in the preoperative period. At discharge, 29 patients remained at level 0, one at level 1, and one at level 2. At the six-month follow-up, all patients were back at level 0, indicating complete functional recovery.

Grade II

Twenty-two patients with grade II AVM were evaluated. On the preoperative GCS, 17 patients were in level I and five in level II. At hospital discharge, 16 patients remained in level I, two in level II, and four had decreased to level III. At the six-month evaluation, all patients (100%) had recovered to level I, with no evidence of residual neurological deterioration.

Regarding the mRS, in the preoperative period, most patients were at levels between 0 and 2 (three at level 0, 12 at level 1, six at level 2, and one at level 3). At discharge, partial improvement in functionality was observed: 15 patients were at level 1, two at level 2, four at level 3, and one at level 4. At six months, 16 patients were at level 0, five at level 1, and one at level 2, reflecting an overall favorable functional evolution as shown in Table 6.

Grade III

Thirty-two patients with grade III AVMs were included. At preoperative evaluation using the GCS, 24 patients were in level I, five in level II, two in level III, and one in level IV. At hospital discharge, 18 patients remained in level I, eight in level II, five in level III, and one in level V. At the six-month follow-up, 30 patients had again reached level I, while one remained in level III and one in level V.

Regarding the mRS, before surgery, 23 patients were at level 1, five at level 3, two at level 2, and one at level 0. At discharge, 18 patients were at level 1, four at level 2, and seven at level 3. At six months, functional improvement was observed in most cases: 21 patients were at level 0, eight at level 1, one at level 2, and one at level 4, as shown in Table 6.

Grade IV

Fourteen patients with grade IV AVMs underwent surgery. Preoperative assessment using the GCS revealed that six patients were in level I, five in level II, two in level III, and one in level IV. At hospital discharge, three patients remained in level I, five in level II, four in level III, and two in level V. At the six-month follow-up, nine patients had reached level I, two were in level II, and three remained in level V.

Regarding the mRS, in the preoperative period, six patients were at level 1, four at level 2, two at level 3, and one at level 4. At discharge, four patients were at level 1, one at level 2, five at level 3, and one at level 4. At six months, limited functional recovery was observed: only three patients were at level 0, six at level 1, two at level 3, and three at level 4, indicating a lower degree of functional recovery compared to the groups with less surgical complexity, as shown in Table 6.

Grade V

Two patients with grade V AVM underwent surgery. In the preoperative evaluation using the GCS, both patients were at level III, maintaining this score both at hospital discharge and at six-month follow-up.

Regarding the mRS, both patients were at level 4 before surgery and did not show significant clinical improvement. They remained at level 4 upon hospital discharge, and at six months, neither had recovered sufficient function to improve their functional scale, as shown in Table 6.

To evaluate functional differences between the groups defined by the SM scale, the nonparametric Kruskal-Wallis test was applied, considering a p value of <0.05 as statistically significant. This test was selected due to the ordinal nature of the functional scales used (Glasgow and Rankin) and the nonnormal distribution of the data.

A comparative statistical analysis using the Kruskal-Wallis test (Table 7) demonstrated no significant difference in GCS scores across SM grades (H = 6.30, p = 0.178), whereas the mRS showed significant functional differences among groups (H = 41.83, p < 0.001), implying that no relationship existed between postsurgery neurological performance and the grade of AVM. Such statistics indicate that, although there are anatomical and operating differences disassociating each grade, the majority of patients still reached a certain state of consciousness comparable to one another.

**Table 7:** Statistical comparison of functional outcomes across Spetzler-Martin grades NS: not significant

Functional measure	H statistic	df	p value	Interpretation
Glasgow Coma Scale (6 months)	6.30	4	0.178	NS (no difference)
Modified Rankin Scale (6 months)	41.83	4	<0.001	Significant difference

On the other hand, a marked difference in functional capacity was found at the end of six months. Use Kruskal-Wallis for independent groups by grade and Friedman for repeated measures within the same group across time. If you conducted multiple tests, present them clearly and correct the description. The patients with the SM grades I and II resulted in essentially full recovery or minimal residual disability, and the patients with the grades IV and V had a stubborn continuation of the functional sequelae.

Follow-up (five years) failed to observe any recurrences of the operated AVMs, and no rebleeding incidences were recorded in the operated group. Moreover, it did not record any death cases due to the malformations themselves or due to intraoperative difficulties throughout the follow-up period.

## Discussion

Although microsurgical outcomes for AVMs have been widely published, long-term data from Latin-American populations are scarce. This study contributes a 100-patient, 12-year multicenter dataset with five-year follow-up, adding region-specific surgical evidence and demonstrating zero recurrence, which is not commonly reported in similar cohorts. The findings of this study reaffirm the predictive value of the SM classification in the clinical and functional outcome of patients with surgically treated cerebral AVMs. Our cohort, with SM I and II recovered well, SM III with multiple outcomes, and SM IV and V with no recovery at all, is consistent with the risk gradient. Other neurosurgical literature supports that SM IV and V (with no recovery) represent the lowest functional independence, and, therefore, the greatest risk reinforces the SM grade’s relevance to recovery. These findings agree with reports from specialized centers reporting microsurgical cure rates for low-grade AVMs of up to 90%-95%, with low rates of permanent neurological deficit [9,12-14].

In patients with grade III AVM, although the complete resection rate was high, functional recovery was somewhat slower, which is consistent with reports indicating greater prognostic variability in this intermediate group [15-17]. The literature highlights that, in these cases, the surgical approach should be individualized and supported by advanced neuroimaging tools and multimodal planning [7,18].

Grade IV and V AVMs showed a significant decrease in functional outcomes and higher mortality (four deaths in total), corresponding to other studies [19,20]. These figures reflect the neurosurgical complexity and high risk associated with resection of high-grade AVMs. Multicenter studies have documented that, in these cases, the combination of therapies (prior embolization, radiosurgery, and staged surgery) can reduce morbidity and mortality [4,19,21]. However, even with advanced techniques, high-grade AVMs remain a therapeutic and ethical challenge for treating teams [7,22].

On the other hand, a higher incidence of AVMs was observed in the right hemisphere and in parietal and frontal regions [20-24]. Furthermore, the prevalence of presentation with seizures (61.4%) and headache (94%) was similar to that described in contemporary cohort studies [22,23].

This study adds to the evidence supporting the efficacy and safety of microsurgical resection for selected AVMs, especially when performed in institutions with neurovascular expertise, precise preoperative planning, and close clinical follow-up [25]. However, it must be recognized that decision-making in AVMs should be guided by a comprehensive risk-benefit analysis, preferences [24].

The results of statistical analysis using the Kruskal-Wallis test revealed significant functional differences in the mRS between the different SM grades, but not in the GCS. This finding is consistent with previous studies that have shown that, although many patients can recover adequate levels of postoperative neurological consciousness (reflected in high Glasgow Coma scores), long-term functional recovery, especially in terms of independence, is significantly influenced by the anatomical and technical complexity of the AVM [9].

Evidence suggests that patients with grade I and II AVMs tend to have minimal or no disability after surgical resection, whereas those with grades IV and V face higher rates of persistent functional dependence [5,19]. This pattern was also observed in our study, where higher grades were associated with Rankin scores above 3 at six-month follow-up. These results reaffirm the prognostic utility of the SM classification and support the need for highly individualized surgical strategies for more complex lesions [20].

Several multicentric and unicentric studies published after our research support and provide context for our findings. For instance, a 2023 research on a single center reported the outcomes of patients after microsurgery and indicated that low-grade lesions had good outcomes, while high preoperative disability and old age were independent predictors of poor outcomes, which emphasizes the need for selecting the patients for surgery carefully [26].

Moreover, some studies on timing of the resection of ruptured AVMs concluded that, although both early and late microsurgical intervention has similar beneficial long-term outcomes, there is also some evidence that high obliteration rates of the AVM may be achieved if the timing of late intervention is carefully planned, which demonstrates the significance of customized timing and the ischemic reservoir in complex cases [27].

The long-term follow-up data also exposed the low, albeit present, risk of re-resection after surgical treatment. In recent works, patients who had undergone surgical treatment presented data on the rates of re-resection that highlighted the importance of angiographic follow-up and prolonged observation, particularly in younger patients. This also resonated with our practice of angiographic and clinical follow-up to document the patients’ durable cure [28].

Taken together, these contemporary series corroborate our main conclusions: microsurgical resection continues to offer excellent obliteration and functional outcomes for low- to medium-grade AVMs when performed in experienced centers, while high-grade lesions require individualized multimodal strategies and extended follow-up. Integrating the present results with these recent reports strengthens the external validity of our findings and highlights priorities for future multicenter and prospective investigations.

Limitations

The present study has some limitations. The procedures were performed by a few neurosurgeons only; therefore, the sample used is narrowed down to match their technique of doing an operation, which may create a bias effect of the operator and prevent generalization. It is recognized that even though data were established prospectively, not all patients were followed up in the long run, and this situation can overestimate the number of delayed complications as well as recurrence. Moreover, not all imaging and intraoperative technologies were the same, as they were applied in the participating centers, and this variable may also affect surgical outcome.

Future directions and clinical implications

Complementary topographical and hemodynamic parameters, such as nidus compactness, venous drainage patterns, and eloquent cortex mapping, should be included in future research to improve surgical risk classification in existing algorithms. To demonstrate the validity of such efforts, multicenter future research on uniform imaging procedures and surgical approaches should be conducted. In addition, research comparing microsurgery to radiosurgery or multimodal treatment, particularly in grade III lesions, would help to determine the therapeutic paradigm.

The current study supports the hypothesis that low-grade AVMs (SM I-II) can be treated with successful functional outcomes and minimum morbidity with microsurgical resection in competent neurovascular centers. Due to clinical variability, a multidisciplinary thorough assessment is critical for intermediate-grade (grade III) AVMs. The absence of recurrences or rebleeding after a long-term follow-up demonstrated the curative effect of complete surgical excision in appropriately selected cases. These findings continue to demonstrate the SM grading system's usefulness as a tool for surgical decision-making.

## Conclusions

Microsurgical excision is another safe and successful treatment for cerebral AVM with SM ratings I and II, resulting in complete functional recovery and little long-term morbidity. The diverse results, despite generally satisfactory outcomes for SM III malformations, highlight the importance of individualized surgical planning and a multidisciplinary assessment of patients. Malformations IV and V in SM, on the other hand, have a higher complication rate and fewer recovery prospects; therefore, extra attention should be made to each patient's risk-benefit ratio when developing a treatment plan.

This study contributes additional outcome data from a Latin-American multicenter experience, supporting the global evidence that microsurgical management achieves the most favorable outcomes in low-grade AVMs, highlighting the necessity of careful selection and multidisciplinary planning, particularly for intermediate and high-grade lesions.

Neurovascular centers with built-in imaging tools, multidisciplinary management, and follow-up provide the most reliable results. Verification of the microsurgical resection's safety and curative potential occurs during long-term maintenance if no recurrence or recurrent bleeding happens, or if the death rate as a result of the surgery occurs during the five-year follow-up period.

## References

[1] Schmidt VF, Masthoff M, Czihal M (2021). Imaging of peripheral vascular malformations - current concepts and future perspectives. Mol Cell Pediatr.

[2] Sabayan B, Lineback C, Viswanathan A, Leslie-Mazwi TM, Shaibani A (2021). Central nervous system vascular malformations: a clinical review. Ann Clin Transl Neurol.

[3] Al-Shahi R, Warlow C (2001). A systematic review of the frequency and prognosis of arteriovenous malformations of the brain in adults. Brain.

[4] Mohr JP, Parides MK, Stapf C (2014). Medical management with or without interventional therapy for unruptured brain arteriovenous malformations (ARUBA): a multicentre, non-blinded, randomised trial. Lancet.

[5] Africk BN, Heiferman DM, Wozniak AW (2021). Angioarchitectural features amongst patients with unruptured brain arteriovenous malformations presenting with headache: findings from a single center retrospective review of 76 patients. J Headache Pain.

[6] Florian IA, Beni L, Moisoiu V, Timis TL, Florian IS, Balașa A, Berindan-Neagoe I (2021). 'De novo' brain AVMs-hypotheses for development and a systematic review of reported cases. Medicina (Kaunas).

[7] Söderman M, Andersson T, Karlsson B, Wallace MC, Edner G (2003). Management of patients with brain arteriovenous malformations. Eur J Radiol.

[8] Spetzler RF, Martin NA (1986). A proposed grading system for arteriovenous malformations. J Neurosurg.

[9] Lawton MT, Rutledge WC, Kim H (2015). Brain arteriovenous malformations. Nat Rev Dis Primers.

[10] Wang M, Jiao Y, Zeng C (2021). Chinese Cerebrovascular Neurosurgery Society and Chinese Interventional & Hybrid Operation Society, of Chinese Stroke Association clinical practice guidelines for management of brain arteriovenous malformations in eloquent areas. Front Neurol.

[11] Cao Y, Zhu W, Shi H, Tie Y, Wang J. (Eds.) (2022). The Clinical Management of Cerebrovascular Disease in Precision Medicine Era. Frontiers Media SA.

[12] Abecassis IJ, Xu DS, Batjer HH, Bendok BR (2014). Natural history of brain arteriovenous malformations: a systematic review. Neurosurg Focus.

[13] Hartmann A, Stapf C, Hofmeister C (2000). Determinants of neurological outcome after surgery for brain arteriovenous malformation. Stroke.

[14] van Swieten JC, Koudstaal PJ, Visser MC, Schouten HJ, van Gijn J (1988). Interobserver agreement for the assessment of handicap in stroke patients. Stroke.

[15] Hellmann F, Marceau E, Cruz R (2024). 60th anniversary of the Declaration of Helsinki: ethical challenges in the 10th amendment. J R Soc Med.

[16] Qian X, Ning W, Liu D, Qu Y, Zhang H (2025). Surgical timing and approach for brainstem cavernous malformation warranting thorough preoperative evaluation. Sci Rep.

[17] Hassan T, Refaat M, Issa AM, Sultan A, Ibrahim T (2023). Geometrical characteristics of grade III arteriovenous malformations that contribute to better outcomes in endovascular treatment. World Neurosurg.

[18] De Leacy R, Ansari SA, Schirmer CM, Cooke DL, Prestigiacomo CJ, Bulsara KR, Hetts SW (2022). Endovascular treatment in the multimodality management of brain arteriovenous malformations: report of the Society of NeuroInterventional Surgery Standards and Guidelines Committee. J Neurointerv Surg.

[19] Li N, Yan D, Li Z (2022). Long-term outcomes of Spetzler-Martin grade IV and V arteriovenous malformations: a single-center experience. Neurosurg Focus.

[20] Miron I, Pruna VM, Visarion DM, Petrescu GED, Gorgan RM (2024). Surgical outcomes and risk factors for overall mortality in brain arteriovenous malformations patients: a retrospective analysis. Front Neurol.

[21] Letchuman V, Mittal AM, Gupta HR (2023). The era of Onyx embolization: a systematic and literature review of preoperative embolization before stereotactic radiosurgery for the management of cerebral arteriovenous malformations. World Neurosurg.

[22] Lauren C, Niryana IW, Mahadewa TG (2025). Impact of embolization on stereotactic radiosurgery outcomes for intracranial arteriovenous malformations Spetzler-Martin grades III-V: a systematic review and meta-analysis. Front Surg.

[23] Deng X, Wang B, Zong F (2021). Right-hemispheric language reorganization in patients with brain arteriovenous malformations: a functional magnetic resonance imaging study. Hum Brain Mapp.

[24] Deng X, Yin H, Zhang Y (2022). Impairment and plasticity of language-related white matter in patients with brain arteriovenous malformations. Stroke.

[25] Sattari SA, Shahbandi A, Yang W, Feghali J, Xu R, Huang J (2023). Microsurgery versus microsurgery with preoperative embolization for brain arteriovenous malformation treatment: a systematic review and meta-analysis. Neurosurgery.

[26] Maalim AA, Zhu M, Shu K (2023). Microsurgical treatment of arteriovenous malformations: a single-center study experience. Brain Sci.

[27] Zhang Y, Chen Y, Han H (2024). Timing of microsurgical resection for ruptured brain arteriovenous malformations: a propensity score-matched analysis using prospective single-center registry data. J Neurosurg.

[28] Järvelin P, Pekonen H, Koivisto T, Frösen J (2023). Recurrence of arteriovenous malformations of the brain after complete surgical resection. Kuopio University Hospital experience and systematic review of the literature. Neurosurg Rev.

